# Autophagy sensitivity of neuroendocrine lung tumor cells

**DOI:** 10.3892/ijo.2013.2136

**Published:** 2013-10-11

**Authors:** SEUNG-KEUN HONG, JIN-HWAN KIM, DMYTRO STARENKI, JONG-IN PARK

**Affiliations:** Department of Biochemistry, Medical College of Wisconsin, Milwaukee, WI 53226, USA

**Keywords:** autophagy, neuroendocrine lung cancer, AKT, mTOR

## Abstract

Neuroendocrine (NE) phenotypes characterize a spectrum of lung tumors, including low-grade typical and intermediate-grade atypical carcinoid, high-grade large-cell NE carcinoma and small cell lung carcinoma. Currently, no effective treatments are available to cure NE lung tumors, demanding identification of biological features specific to these tumors. Here, we report that autophagy has an important role for NE lung tumor cell proliferation and survival. We found that the expression levels of the autophagy marker LC3 are relatively high in a panel of lung tumor cell lines expressing high levels of neuron-specific enolase (NSE), a key NE marker in lung tumors. In response to bafilomycin A1 and chloroquine, NE lung tumor cells exhibited cytotoxicity whereas non-NE lung tumor cells exhibited cytostasis, indicating a distinct role of autophagy for NE lung tumor cell survival. Intriguingly, in certain NE lung tumor cell lines, the levels of processed LC3 (LC3-II) were inversely correlated with AKT activity. When AKT activity was inhibited using AKTi or MK2206, the levels of LC3-II and SQSTM1/p62 were increased. In contrast, torin 1, rapamycin or mTOR knockdown increased p62 levels, suggesting that these two pathways have opposing effects on autophagy in certain NE lung tumors. Moreover, inhibition of one pathway resulted in reduced activity of the other, suggesting that these two pathways crosstalk in the tumors. These results suggest that NE lung tumor cells share a common feature of autophagy and are more sensitive to autophagy inhibition than non-NE lung tumor cells.

## Introduction

Lung cancer is currently the leading cause of cancer death in men and women, causing more deaths than colon, breast and prostate cancer combined (1). Lung cancers are mainly classified into small cell lung carcinoma (SCLC) and non-small cell lung carcinoma (NSCLC), but lung cancers are also sub-classified depending upon different characteristics and origins of progenitor cells (2–4). For example, neuroendocrine (NE) phenotypes characterize a spectrum of tumors, including low-grade typical and intermediate-grade atypical carcinoid, high-grade large-cell NE carcinoma and SCLC (5,6). NE lung tumors comprise 20–25% of all invasive lung malignancies. Among those, SCLC is the most common pulmonary NE tumor which accounts for 15–20% of invasive lung malignancies whereas carcinoid tumors and large-cell NE carcinoma represent minor fraction of invasive lung malignancies (5,6). Currently, no effective treatments are available to cure SCLC and other NE lung tumors and it is necessary to identify a biological feature specifically involved in growth and survival of these tumor cells.

Autophagy is an important cellular recycling process to overcome limited availability of nutrients, which is mediated by a constitutive lysosomal degradation pathway (7). Under stress conditions, autophagy is upregulated to generate resources for the maintenance of essential cellular functions (8,9). However paradoxically, autophagy can also trigger cell death under certain conditions. Therefore, a balance between these two opposing effects of autophagy influences cellular differentiation, development, homeostasis and the development of different diseases, including cancer (7). A key step in mediating autophagy is the formation of autophagosomes, which is mediated by microtubule-associated protein-1 light chain-3 (LC3), a mammalian homolog of yeast autophagy-related gene 8 and the LC3 binding protein, SQSTM1/p62 (10). During autophagy, the cytoplasmic form of LC3 (LC3-I, 18 kDa) is recruited to the autophagosomes, where LC3-II (16 kDa) is generated by site-specific proteolysis and lipidation near to the C-terminus (11). Therefore, autophagic activity is measured biochemically as the amount of LC3-II and p62 that accumulates in the absence or presence of lysosomal activity.

Recent studies have demonstrated the significance of autophagy in pulmonary epithelial cell proliferation and survival. For example, it has been demonstrated that increased autophagy contributes to the pathogenesis of chronic obstructive pulmonary disease by promoting epithelial cell death (12). It has also been shown that LC3 confers protection against hypoxia-induced pulmonary hypertension (13). Nevertheless, the significance of autophagy in lung cancer is yet unclear. Further, no studies in the context of autophagy in NE lung tumors have been reported.

Here, we demonstrate that, in a panel of human lung cancer lines, steady-state levels of the autophagy marker, LC3, are relatively high in the cell lines expressing neuron-specific enolase (NSE), a key NE marker in lung tumor (14,15). We then show that those cell lines are more sensitive to autophagy inhibitors than non-NE lung tumor cells. We also investigate the involvement of AKT and mammalian target of rapamycin (mTOR) pathways, the two known regulators of autophagy (16,17), in autophagy regulation in these cells.

## Materials and methods

### Cell culture and reagents

The human lung cancer lines, DMS53, NCI-H209, NCI-H82, NCI-H69, NCI-H889, NCI-H345, SHP-77, A549, NCI-H23, NCI-H460, NCI-H1155, NCI-H358, NCI-H727, NCI-H125 and NCI-H1770 (ATCC), were maintained in phenol red-deficient RPMI-1640 (Invitrogen, Carlsbad, CA, USA) supplemented with 10% fetal bovine serum (FBS), 100 U of penicillin and 100 *μ*g of streptomycin per ml. Bafilomycin A1 and chloroquine were purchased from Sigma (St. Louis, MO, USA). Torin 1, rapamycin, AKTi and MK-2206 were purchased from TOCRIS (Minneapolis, MN, USA), Cell Signaling Technology (Danvers, MA, USA), EMD Chemicals Inc. (Chicago, IL, USA) and Active Biochem (Maplewood, NJ, USA), respectively.

### Small hairpin RNA (shRNA) expression construct

The lentiviral pLKO.1-shRNA vector targeting mTOR (Addgene plasmid 8454) was previously described (18). For lentivirus production, 293T cells were co-transfected with pLKO.1 and packaging vectors, as previously described (19,20). Viral supernatants were collected after 48–72 h and mixed with polybrene (Sigma) at 4–8 *μ*g/ml before use. Viral titer was determined by scoring cells resistant to puromycin.

### Cell survival and cell cycle assays

To determine cell survival rates, cells were seeded in 24-well plates (Corning, Corning, NY, USA) at a density of 5,000 cells per well. Cell proliferation and death was determined by counting trypan blue-stained cells using hemocytometer. For cell cycle analysis, cells were washed with ice-cold 0.2% BSA in PBS, resuspended in 250 mM sucrose/40 mM citrate buffer (pH 7.6) containing 0.5% DMSO. Nuclei were prepared, stained with propidium iodide (21) and analyzed by LSR II Flow Cytometer (Becton-Dickinson, Franklin Lakes, NJ, USA) with a gate that selects single nuclei within a normal size range. The cell cycle parameters from 10,000 gated nuclei were determined and subsequent analysis was conducted using FCS Express software (De Novo software, Los Angeles, CA, USA).

### Immunoblot analysis

Cells harvested at various times were lysed and analyzed for protein concentration using the BCA reagent (Pierce, Rockford, IL, USA), as previously described (22). Protein (50–100 μg) was resolved by SDS-PAGE, transferred to a polyvinylidene difluoride membrane filter (Bio-Rad, Hercules, CA, USA) and stained with Fast Green reagent (Thermo Fisher Scientific, Waltham, MA, USA). Membrane filters were then blocked in 0.1 M Tris (pH 7.5)/0.9% NaCl/0.05% Tween-20/5% non-fat dry milk and incubated with appropriate antibodies. Antibodies were diluted as follows: NSE, 1:2,500; PARP, 1:1000 (Thermo Fisher Scientific); AKT, 1:2,500; phospho-AKT (Ser473), 1:5,000; phospho-GSK-3β (Ser9), 1:2,500; phospho-ERK1/2 (Thr202/Tyr204), 1:2,500; GAPDH, 1:5,000; phospho-p70S6K1 (Thr389), 1:2,000; phospho-mTOR (Ser2481), 1:2,000; phospho-4E-BP1 (S65), 1:1,000; phospho-S6 (S235/236), 1:1,000 (Cell Signaling Technology); p62, 1:2,000 (Santa Cruz Biotech, Santa Cruz, CA, USA); LC3B, 1:2,000 (MBL International, Woburn, MA, USA). The Supersignal West Pico and Femto chemiluminescence kits (Pierce) were used for visualization of the signal. Immunoblots were scanned and analyzed using Image Lab (Bio-Rad).

## Results

### The levels of LC3-I are upregulated in NE lung tumor cell lines

**Figure 1.**
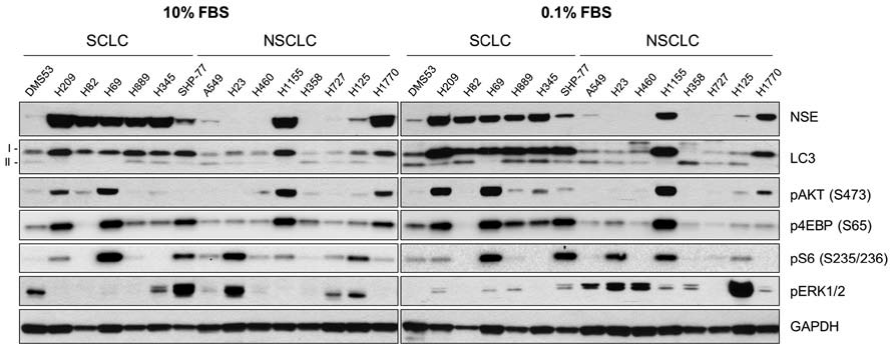
LC3 levels are upregulated in human NE lung tumor cell lines. Total lysates of SCLC and NSCLC cells maintained in RPMI-1640 containing 10 or 0.1% FBS for 2 days were analyzed by western blotting for NSE expression, LC3 processing (LC3-1 to LC3-II) and phosphorylation of AKT (pAKT), 4E-BP (p4EBP), the ribosomal protein S6 (pS6) and ERK1/2 (pERK1/2). Glyceraldehyde-3-phosphate dehydrogenase (GAPDH) was detected to validate equal protein loading.

To profile steady-state levels of autophagy in different lung tumor types, we analyzed LC3 levels in 15 human lung tumor cell lines, including 7 SCLC and 8 NSCLC cell lines. In this panel, cell lines were also examined for NSE expression as a marker of NE phenotype. All SCLC cell lines except for DMS53 expressed high levels of NSE whereas among the NSCLC cell lines, only the known NE lines, NCI-H1155 and NCI-H1770 (23), expressed similarly high levels of NSE (Fig. 1).

In this cell line panel, we detected relatively high LC3-I levels in strong correlation with high NSE levels (Fig. 1). Because LC3-II levels were not clearly detected under the culture condition using 10% FBS (Fig. 1, left panel), we also analyzed cells maintained in the media containing 0.1% FBS, which would increase the cellular demand for autophagy (Fig. 1, right panel). Indeed, the formation of LC3-II became prominent in most cell lines under this serum-starved culture condition (Fig. 1, right panel). However, this condition did not increase LC3-II formation in the NSE expressing cell lines, NCI-H209, NCI-H69, NCI-H1155 and NCI-H1770 cells (Fig. 1, right panel) and very intriguingly, this phenomenon was correlated with relatively high basal levels of AKT phosphorylation at Ser473, an indication of AKT activation (24) (Fig. 1).

In this cell line panel, we also examined the mTOR and ERK1/2 pathways, which are often deregulated in cancer. However, neither ERK1/2 activity, as indicated by phosphorylation of their activation loop (Thr202/Tyr204 of ERK1 and Thr183/Tyr185 of ERK2), nor activity of the mTOR pathway, as indicated by phosphorylation of 4E binding protein 1 (4E-BP) and the ribosomal protein S6, were significantly correlated with the low LC3-II levels (Fig. 1, right panel). 4E-BP is phosphorylated by mTOR and S6 is a substrate of S6 kinase 1 (S6K1), which is also phosphorylated by mTOR (25). These data demonstrate a strong correlation between LC3 and NSE levels, suggesting a potential significance of autophagy in NE lung tumor cells. These data also suggested a potential involvement of AKT in autophagy regulation in certain NE lung tumor types.

### NE lung tumor cells are more sensitive to autophagy inhibitors than non-NE lung tumor cells

**Figure 2.**
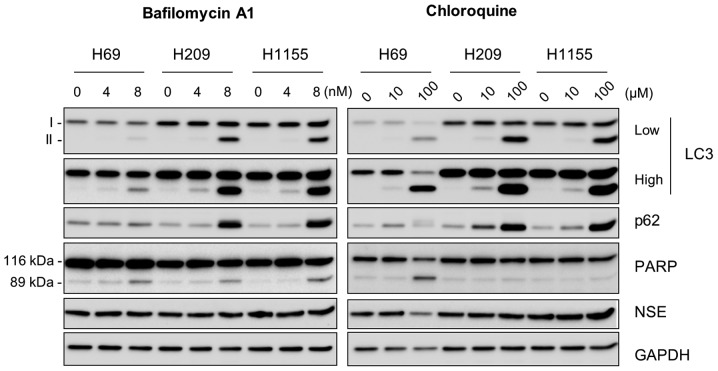
Autophagy inhibitors induce PARP cleavage in NE lung tumor cells. Cells were treated with increasing doses of the autophagy inhibitors, bafilomycin A1 and chloroquine, in RPMI-1640 containing 10% FBS for 2 days. Equivalent volume of dimethyl sulfoxide was used as the vehicle control. LC3 processing, p62 accumulation, PARP cleavage and NSE expression were determined by western blot analysis of total cell lysates. GAPDH was the control for protein loading.

To determine the significance of autophagy in NE lung tumor cells, we examined the effects of inhibition of steady-state autophagy in NCI-H69, NCI-H209 and NCI-H1155 cells using bafilomycin A1 and chloroquine, the lysosomotropic agents that inhibit lysosomal degradation of autophagosome. As expected, these cells exhibited highly increased LC3-II and p62 levels within 48 h in response to the inhibitor treatment (Fig. 2), which indicates the accumulation of these proteins due to delayed autophagy. Of note, bafilomycin A1 significantly increased cleavage of poly(ADP-ribose) polymerase (PARP), an indication of caspase-dependent apoptosis, in all these cell lines, although chloroquine increased PARP cleavage only in NCI-H69 cells (Fig. 2). In contrast, NSE levels were unaffected under these conditions except that chloroquine decreased NSE levels in NCI-H69 cells (Fig. 2, right panel). These data suggest the importance of autophagy for survival, but not for NE phenotype, of NE lung tumor cells.

**Figure 3.**
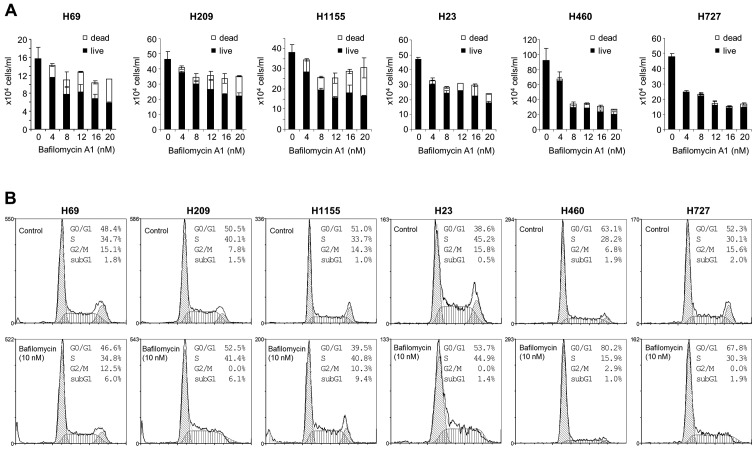
Autophagy inhibition induces cell death in NE lung tumor cells but not in non-NE lung tumor cells. NE lung tumor cell lines (NCI-H69, NCI-H209 and NCI-H1155) and non-NE lung tumor lines (NCI-H23, NCI-H460 and NCI-H727) were treated with increasing doses of bafilomycin A1 in RPMI-1640 containing 10% FBS for 2 days. (A) Cell viability was determined by scoring trypan blue-stained cells. (B) Cell cycle analysis was carried out using cells treated with 10 nM bafilomycin A1 for 2 days. Data (mean ± standard error) are from a representative experiment performed in triplicate.

Subsequently, we analyzed NCI-H69, NCI-H209 and NCI-H1155 cells in comparison with the non-NE lung tumor cell lines, NCI-H23, NCI-H460 and NCI-H727 for their sensitivity to different doses of bafilomycin A1. We found that bafilomycin A1 could effectively suppress proliferation of these tumor lines regardless of NE phenotypes (Fig. 3A). However intriguingly, the mechanisms of growth inhibition appeared quite different between NE and non-NE lung tumor lines. Bafilomycin A1 treatment increased cell death more significantly in NCI-H69, NCI-H209 and NCI-H1155 cells than in NCI-H23, NCI-H460 and NCI-H727 cells, as determined by trypan blue staining (Fig. 3A). Consistent with this, bafilomycin A1 significantly increased sub-G1 phase cell populations in NCI-H69, NCI-H209 and NCI-H1155 cells whereas the drug induced G0/G1 phase arrest, but did not increase sub-G1 population, in NCI-H23, NCI-H460 and NCI-H727 cells (Fig. 3B). Increases in sub-G1 phase population indicate onset of programmed cell death and thus, accord with increased trypan blue staining (Fig. 3A) and PARP cleavage (Fig. 2, left panel) in bafilomycin A1-treated NCI-H69, NCI-H209 and NCI-H1155 cultures. These data suggest that autophagy inhibition can induce different growth inhibitory effects in different lung tumor types, i.e., cytotoxicity in NE lung tumor types versus cytostasis in non-NE lung tumor types.

### AKT and mTOR pathways regulate autophagy in certain NE lung tumor types in an opposing context

**Figure 4.**
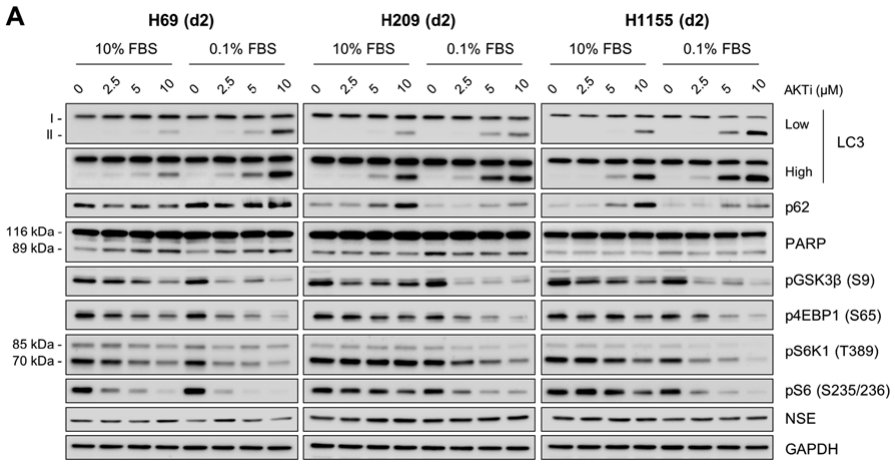

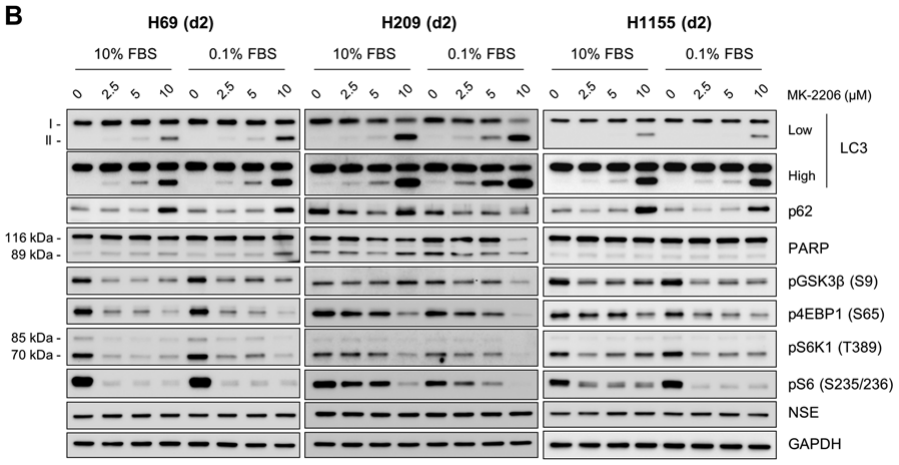
AKT inhibitors induce similar effects on LC3 and p62 as autophagy inhibitors in NE lung tumor cells. Cells were treated with increasing doses of the AKT inhibitor, AKTi (A) or MK-2260 (B), in RPMI-1640 containing 10% FBS for 2 days. LC3 processing, p62 accumulation, PARP cleavage, GSK3β phosphorylation, mTOR pathway activity (indicated by phosphorylation of 4E-BP, S6K1 and S6) and NSE expression were analyzed by western blotting of total cell lysates. GAPDH was the control for protein loading.

The correlation between low LC3-II levels and high AKT phosphorylation in NCI-H69, NCI-H209 and NCI-H1155 cells (Fig. 1) led us to investigate the role of AKT in autophagy regulation in these cells. For this, we examined the effects of AKT inhibition in these cells using the two structurally-unrelated AKT specific inhibitors, AKTi and MK-2206. We found that inhibition of AKT activity, as indicated by decreased phosphorylation of its substrate GSK3β, substantially increased LC3-II levels in these cells regardless of the culture conditions, i.e., 10% versus 1% FBS (Fig. 4). Along with this, p62 levels were also increased in these cells, which was more significant in cells maintained using 10% FBS (Fig. 4). However, NSE expression was not affected by these inhibitors, suggesting that AKT may not affect NE phenotypes of these cells. These effects of AKT inhibition are consistent with the effects of bafilomycin A1 and chloroquine on these NE lung tumor cell lines, suggesting a role for AKT in autophagy regulation in certain NE lung tumor types.

**Figure 5.**
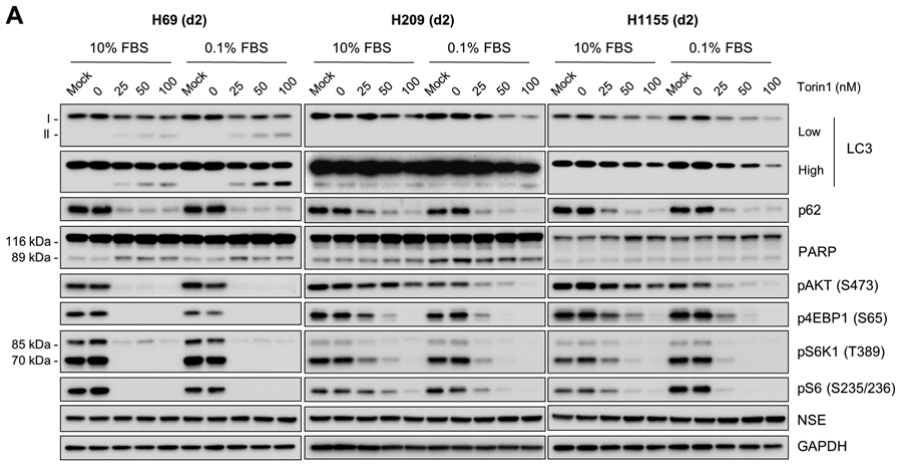

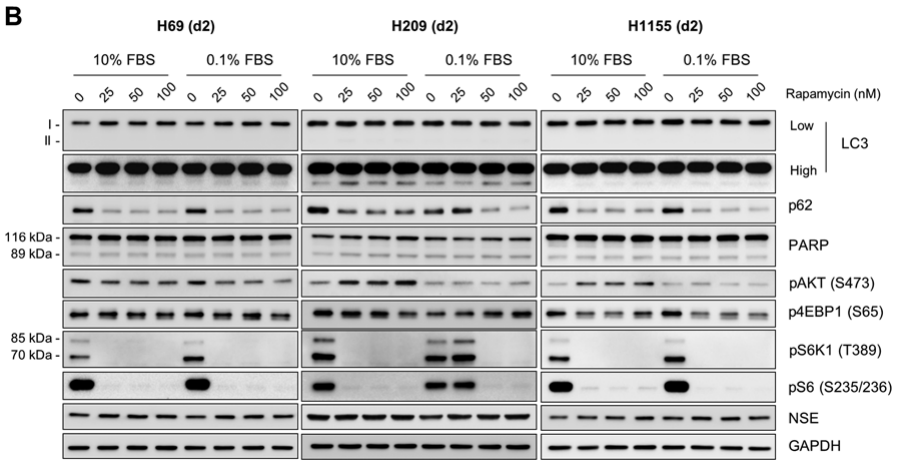
mTOR inhibitors, torin 1 and rapamycin, consistently affect autophagy markers, but not AKT activity, in NE lung tumor cells. Cells were treated with increasing doses of the mTOR inhibitors, torin 1 (A) and rapamycin (B), in RPMI-1640 containing 10% FBS for 2 days. LC3 processing, p62 accumulation, PARP cleavage, AKT phosphorylation and mTOR pathway activity (indicated by phosphorylation of 4E-BP, S6K1 and S6) were analyzed by western blotting of total cell lysates. GAPDH was the control for protein loading.

Since mTOR complex (mTORC) 1 is well known for its antagonizing effect on autophagy (16,17) and because high mTOR pathway activity was also detected in NCI-H69, NCI-H209 and NCI-H1155 cells (Fig. 1), we next investigated the effects of mTOR inhibitors, torin 1 and rapamycin, on LC3 and p62 in these cells. Whereas torin 1 inhibits both mTORC1 and mTORC2, rapamycin inhibits only mTORC1 (26). Both inhibitors effectively inhibited phosphorylation of S6K1 and its substrate S6, although torin 1 inhibited 4E-BP1 phosphorylation more effectively than rapamycin (Fig. 5), indicating significantly reduced mTOR activity in these cells. Under these conditions, p62 levels were significantly decreased in all three cell lines (Fig. 5). No significant increases in LC3-II levels were detected, although torin 1-treated NCI-H69 cells exhibited mild increases, which were still lower than the levels increased by the AKT inhibitors (Fig. 5; compare with Fig. 4). These changes are consistent with the known effects of mTORC1 inhibition to trigger autophagy, leading to p62 depletion (16,17). These opposite effects on p62 of the AKT and mTOR inhibitors suggest that the mTOR and AKT pathways have opposing roles in autophagy regulation in certain NE lung tumor types.

### AKT and mTOR pathways crosstalk in certain NE lung tumor types

AKT can regulate mTORC1 activity via phosphorylation of tuberous sclerosis complex 2 or PRAS40 (27–29). Conversely, mTOR can also regulate AKT activity via mTORC2-mediated phosphorylation of Ser473 in the hydrophobic motif of AKT (18). Given the coincident activation of AKT and mTOR pathways in NCI-H69, NCI-H209 and NCI-H1155 cells, we determined whether these two pathways can crosstalk in these cells by examining the effects of AKT and mTOR inhibitors on the surrogate markers of each pathway.

AKTi and MK-2206 treatments mildly but consistently decreased phosphorylation of 4E-BP1 and S6K1 with the decreases being more significant under the low serum culture condition (Fig. 4), suggesting that AKT can affect activity of the mTOR pathway in these NE lung tumor cells. Whereas torin 1 inhibited AKT phosphorylation in these cell lines (Fig. 5A), rapamycin rather increased AKT phosphorylation in NCI-H209 and NCI-H1155 cells, albeit not in NCI-H69 (Fig. 5B), suggesting that mTORC1 and mTORC2 may antagonistically regulate AKT in certain NE lung tumors.

**Figure 6.**
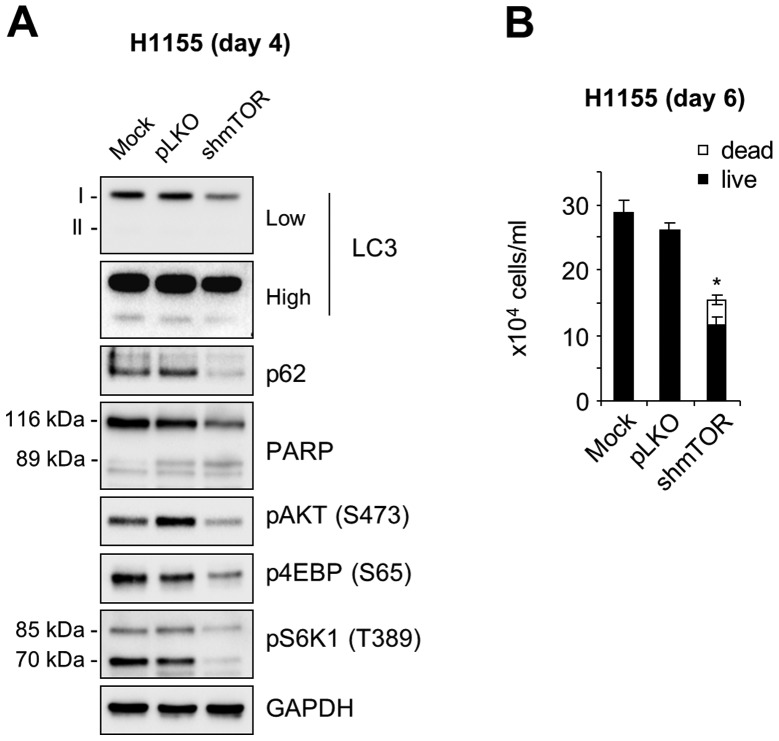
mTOR knockdown downregulates p62 levels and suppresses cell proliferation/survival in NCI-H1155 cells. Cells were infected with the mTOR specific lentiviral shRNA construct (shmTOR) or the control pLKO.1 in 10% FBS-containing medium for the indicated time. (A) Total cell lysates were analyzed by western blotting for expression of the indicated proteins. ^*^p<0.05 versus pLKO for live cells (Student’s t-test). (B) Cell viability was determined by scoring trypan blue-stained cells. Data (mean ± standard error) are from a representative experiment performed in triplicate.

The effect of torin 1, which inhibits both mTORC1 and mTORC2 activity, was confirmed by a lentiviral shRNA construct that was previously used to specifically knockdown mTOR activity in cells (18). Consistent with the effects of torin 1 and rapamycin, mTOR knockdown also reduced the levels of p62, as determined in NCI-H1155 cells (Fig. 6A). Under this condition, AKT phosphorylation was significantly decreased (Fig. 6A), which is consistent with the effect of torin 1. Of note, mTOR knockdown significantly increased cell death and suppressed cell proliferation in NCI-H1155 cultures (Fig. 6B). These data therefore suggest that the AKT and mTOR pathway can antagonistically regulate autophagy in certain NE lung tumor cells and that crosstalk between these pathways may contribute to the regulation of autophagy and cell survival in the tumors.

## Discussion

This study demonstrates that human NE lung tumor cell lines maintain relatively high LC3 levels and sensitivity to autophagy inhibition when compared with non-NE lung cancer types, suggesting that autophagy may have important roles in NE lung tumors.

The striking correlation between LC3 and NSE levels in lung tumor cells leads to a question why LC3 levels are high in NE lung tumors and what advantages it confers to the tumor type. It was previously proposed that autophagy in lung epithelium indicates an adaptive response to stress-induced injury, which is caused by hypoxia, oxidants, inflammation, ischemia-reperfusion, endoplasmic reticulum stress, pharmaceuticals, or cigarette smoke (30). Particularly, chronic exposure to cigarette smoke or cigarette smoke extract increased autophagy in mouse lungs and in pulmonary epithelial cells (12). Further, knockdown of cigarette smoke-induced autophagy mediators inhibited apoptosis *in vitro,* suggesting that autophagy has a role in regulating lung epithelial cell survival (12). Since SCLC is mainly caused by tobacco smoke (2), it is conceivable that the relatively high LC3 levels in SCLC cells may reflect an etiological alteration attributed to tobacco smoke. Additional explanations are also available. A recent study suggests that LC3 confers protection against hypoxia-induced pulmonary hypertension by inhibiting proliferation of pulmonary artery wall cells (13). A similar mechanism may underlie the progression of NE lung tumors. For example, LC3 may confer an advantage for tumor cell survival under a hypoxic condition, which is often associated with the development of solid tumors.

The relatively high sensitivity of the tested NE lung tumor cell lines to autophagy inhibition may present a potential clinical significance. Currently, no effective treatments are available to cure SCLC or other NE lung tumors. Conventionally, SCLC is initially treated by combination chemotherapy using cisplatin or carboplatin plus etoposide with an option to include radiation therapy, which results in overall high response rates (60–80%) (4). However, tumors relapse within months after the initial therapy and topotecan is the only approved agent for recurrent or progressive SCLC (31,32). Accordingly, SCLC patients have a very poor survival of <5% at 5 years (33). Atypical carcinoids and large cell NE carcinomas also pose clinical problems because the optimal therapy for them is not available (5,6). Therefore, there is a significant demand for the development of new therapeutic strategies for SCLC and other NE lung tumors. Autophagy inhibition may be useful to suppress NE lung tumors because autophagy inhibition induces programmed cell death in NE lung tumor cells. Of note, recent studies show that a number of different chemotherapeutic agents induce autophagic alteration as a mechanism underlying their therapeutic effects (34). Indeed, chloroquine has been evaluated in multiple clinical studies of different cancers (34). Our results suggest a careful consideration of these therapeutic modalities in NE lung cancer. In addition, since our study suggests that AKT and mTOR pathways are among the key signaling pathways that regulate autophagy in certain NE lung tumors, it may be possible to target these kinases to disrupt the balance of autophagy in the tumors.

Intriguingly, it has been suggested that NSE expression is associated with the degree of tumor malignancy and, thus, NSE has been proposed as a marker for staging and monitoring of NE lung tumor (14,35,36). Therefore, the strong correlation between NSE and LC3 levels may indicate the possibility that an autophagic alteration underlies NE lung tumor malignancy and that LC3 is a potential prognostic biomarker. Distinct alterations in metabolism and signal transduction might lead to unique biological and clinical features of lung cancer and identification of these alterations could contribute to the development of novel therapeutic strategies. Our study suggests that autophagy may be a unique feature characterizing NE lung tumors.

## Abbreviations:

4E-BP: 4E binding protein 1;
GAPDH: glyceraldehyde 3-phosphate dehydrogenase;
LC3: microtubule-associated protein-1 light chain-3;
mTOR: mammalian target of rapamycin;
mTORC: mTOR complex;
NE: neuroendocrine;
NSE: neuron-specific enolase;
NSCLC: non-small cell lung carcinoma;
S6: ribosomal protein S6;
S6K1: S6 kinase 1;
SCLC: small cell lung carcinoma;
